# Thoracic duct identification with indocyanine green fluorescence to prevent chyle leaks during minimally invasive esophagectomy

**DOI:** 10.1002/cnr2.2053

**Published:** 2024-04-05

**Authors:** Habibollah Mahmoodzadeh, Athena Farahzadi, Ramesh Omranipour, Iraj Harirchi, Amirmohsen Jalaeefar, Mohammad Shirkhoda, Seyed Rouhollah Miri, Farimah Hadjilooei

**Affiliations:** ^1^ Department of General Surgery Tehran University of Medical Sciences, Cancer Institute Tehran Iran; ^2^ Tehran University of Medical Sciences, Cancer Institute Tehran Iran

**Keywords:** chylothorax, indocyanine green, minimally invasive esophagectomy, near‐infrared fluorescence, thoracic duct leaks

## Abstract

**Introduction:**

Chylothorax (CT) is a rare yet serious complication after esophagectomy. Identification of the thoracic duct (TD) during esophagectomy is challenging due to its anatomical variation. Real‐time identification of TD may help to prevent its injury. Near infra‐red imaging with Indocyanine green (ICG) is a novel technique that recently has been used to overcome this issue.

**Methods:**

Patients who underwent minimally invasive esophagectomy for esophageal cancer were divided into two groups with and without ICG. We injected ICG into bilateral superficial inguinal lymph nodes. Identification of TD and its injuries during the operation was evaluated and compared with the non‐ICG group.

**Results:**

Eighteen patients received ICG, and 18 patients underwent surgery without ICG. Each group had one (5.5%) TD ligation. In the ICG group injury was detected intraoperative, and ligation was done at the site of injury. In all cases, the entire thoracic course of TD was visualized intraoperatively after a mean time of 81.39 min from ICG injection to visualization. The Mean extra time for ICG injection was 11.94 min. In the ICG group, no patient suffered from CT. One patient in the non‐ICG group developed CT after surgery that was managed conservatively. According to Fisher's exact test, there was no significant association between CT development and ICG use, possibly due to the small sample size.

**Conclusions:**

This study confirms that ICG administration into bilateral superficial inguinal lymph nodes can highlight the TD and reduce its damage during esophagectomy. It can be a standard method for the prevention of postoperative CT.

## 1 INTRODUCTION

The primary treatment for esophageal cancer involves a combination of esophagectomy and preoperative or postoperative chemotherapy and radiotherapy.^1^ Minimal invasive esophagectomy is commonly used nowadays and offers proven benefits such as reduced surgical invasiveness and improved visibility of anatomical structures. Even with this advancement, the procedure still has postoperative complications as high as 59%.^1^ Thoracic duct (TD) injury is a serious but uncommon complication of esophagectomy. It leads to chylothorax (CT) in 1%–9% of the patients. It delays oral intake, lengthens hospital stays, lead to respiratory complications in frail patients and reduce overall survival rate, subjects a frail patient to an extrathoracotomy or thoracoscopy with possible respiratory complications, reduces overall survival, and increases mortality rates by up to 20%.^2^, ^3^ The best management strategy for CT is still unclear. Initial conservative treatment is usually applied in cases with <1 L per day output from the thoracic drain. The treatment involves nil per os, total parenteral nutrition, and drainage of the thorax, with or without the use of medications like somatostatin or octreotide.

An invasive approach is usually mandatory when the output surpasses 1 L per day or conservative treatment fails.^3^ In case of a leak, surgical ligature of the TD during reoperation surgery has a success rate of over 90% and seems mandatory, despite being a challenging procedure.^3^

If the injury site is identified, direct ligation with a nonabsorbable suture should be adopted.

If the site of injury cannot be located, the preferred method is to perform supradiaphragmatic ligation between the descending thoracic aorta and azygos vein. This procedure involves blocking the duct at its entry into the thorax and prevents the chyle leakage from the accessory ducts. This procedure is usually performed utilizing thoracoscopy, which magnifies the image and facilitates the identification of the chyle leak site.^4^

Intraoperative identification of TD is the best prophylactic means to avoid injury or timely managed damage when it happens.^2^ TD anatomy is widely variable, which complicates intraoperative identification of the TD and its leakage site.^5^ Oral administrations of heavy cream or oil before surgery are traditionally used to identify the TD and decrease its iatrogenic injury. lymphangiography and lymphoscintigraphy can reconstruct the anatomy of TD preoperatively, although transferring to the operative field is troublous and is not routinely used.^2^ Currently, there are no standard and effective intraoperative diagnostic tools for visualization of TD. However, our team used ICG fluorescence to accurately identify the TD in real‐time.

Invisible near‐infrared (NIR) fluorescence imaging, which utilizes a fluorescent dye called indocyanine green (ICG), is a new way of visualizing tissue during surgery. The merging of NIR imaging and ICG dye allows for the detection of light emitted by the dye, which is then superimposed on the video image to highlight the area of fluorescence. This technique has proven to be useful in assisting surgeons to better visualize tissues and achieve better outcomes during surgery.^4^ In complicated, recurring, and refractory cases, it helps identify chyle leaks and facilitates the localization of TD injury.^6^ In the literature, ICG fluorescence has been used intraoperatively to detect the TD and its lesions in patients with CT after esophagectomy, neck dissection, or lung surgery. In a few studies, including ours, ICG angiography has been used to visualize the TD during surgery.

Vecchiato et al. used ICG angiography to identify the course of TD during transthoracic esophagectomy (TTE) and successfully identified TD in 95% of cases.

This study is an interventional study using historical controls. We recruited patients with esophageal cancer who were candidates for minimally invasive McKeown esophagectomy. The primary aim was to identify the anatomy of the TD, its intraoperative injury, and appropriate treatment of the damage using NIR fluorescence imaging. We compared the results with patients who underwent a similar procedure without using NIR fluorescence imaging with ICG. This technique has been utilized for the first time in our country. This study is the first to compare the results with the same procedure without ICG.

## 2 MATERIALS AND METHODS

Our study aims to assess the feasibility of using NIR fluorescence‐guided thoracoscopy to detect TD injury, identify its anatomy, and manage it effectively during minimally invasive esophagectomy. We compared the results with a historical control group who underwent minimally invasive esophagectomy without this novel technique.

All patients with esophageal cancer who were candidates for minimally invasive McKeown esophagectomy between September 2020 and June 2022 enrolled in the study. To be included in the study, patients had to be 18 years or older, classified as the American Society of Anesthesiologists (ASA) Class I, II, or III, undergo elective surgery, and provide written consent to participate. Patients with an allergy to iodine, a history of asthma, inability to undergo TTE, pregnancy, and those who were unable to understand the consensus were excluded from the study. The study was approved by the Imam Khomeini Hospital Research Ethics Committee, which is sponsored by the Tehran University of Medical Science. The code of ethics was IR, TUMS.IKHC.REC.14400.413. The imaging device and the ICG compound are FDA‐cleared and routinely used in clinical care. A multidisciplinary expert team formulated the patients' treatment strategies. All patients in the study provided written informed consent.

### 2.1 Surgical techniques

The patient had double‐lumen endotracheal intubation in the supine position. Our surgeon injected 1‐mg ICG into bilateral superficial inguinal lymph nodes (LNs) under ultrasonography guidance. The patient's position changed to Left lateral decubitus. Four trocars were placed in the right hemi thorax; first in the fourth to fifth intercostal space at the midclavicular line and second in the ninth intercostal space at the anterior axillary line, and the third one anterior to the scapular tip. The last trocar was placed posterior to the third one. After one lung ventilation, a right pneumothorax is made at 8 mmHg pressure. The azygous vein was identified and dissected. It was ligated with hemoclips at the arc level. Dissection of the esophagus with periesophageal tissue was performed with harmonic coagulation cranially to the pleural dome and caudally to the hiatus. The TD was visualized by switching to the NIR mode during periesophageal dissection. Precautions were taken not to injure the duct. Mediastinal lymphadenectomy was performed. Tube thoracostomy inserted in right hemi thorax. Laparoscopic gastrolysis was carried out with harmonic and hemoclips after repositioning to supine. Right gastric and gastroepiploic vessels were preserved. The celiac lymph nodes were dissected. After the Left cervicotomy, the cervical esophagus was released. The gastric conduit was created with GIA staplers after upper midline mini‐laparotomy. Pyloromyotomy was performed when our surgeon believed the patient had high‐pressure pylorus. A circular stapler created a cervical end‐to‐side esophagogastrostomy. Tube thoracostomy was inserted into the left hemi thorax if the left pleura opened.

### 2.2 ICG fluorescence near infrared lymphangiography

We injected 10 mg ICG into superficial inguinal lymph nodes bilaterally under ultrasonography guidance. Twenty‐five‐milligram ICG was diluted in 10 ccs sterile water. We used the STRYKER 1588AIM camera system. We can switch from standard mode to NIR mode using the camera button for TD visualization and checking its injury during operation after some dissection of periesophageal tissue; when visualization of the TD was optimal. The operating room light was turned off during imaging.

Identification of the TD, its injury, and proper management of injured TD during the operation were our main goals in this study. We compared the results with a historical control group. Secondary outcomes measured in the study included adverse reactions, pain, iatrogenic lesions, and complications at the injection site. The study collected data on several factors, including sex, age, body mass index, American Society of Anesthesia score, surgical technique, operative time, adverse reactions, iatrogenic lesions, complications, and problems at the injection site, histology, and site of the tumor, neoadjuvant treatment, injection site, the dose of ICG, time from injection to visualization, need for intraoperative ligation of TD, development of CT, hospital stay.

### 2.3 Statistical analysis

Demographic parameters were summarized using descriptive statistics. This study presents the measured data as mean ± standard deviation. To compare groups and determine the difference between the two groups, the independent sample *t*‐test, chi‐square test, and Fisher exact test were used. Statistical analysis was completed using SPSS® version 22, with a *p*‐value <.05 indicating a statistically significant difference.

## 3 RESULTS

### 3.1 Demographic parameters

A total of 36 patients participated in the study, with 18 patients undergoing minimally invasive McKeown esophagectomy with ICG and the remaining 18 undergoing the same procedure without ICG. Table 1 summarizes the characteristics of the patients and tumor. There were no significant differences in age, sex, smoking, ASA, comorbidities, neoadjuvant chemotherapy, or tumor location between the two groups. Squamous cell carcinoma was the most common pathological type in both groups.

**TABLE 1:** Demographic parameters and tumor characteristics of the patients.

	ICG‐group (*n* = 18)	Non‐ICG‐group (*n* = 18)	*p*‐Value
Age (years)	56.44	56.39	.986
Sex			.505
Female	10	8	
Male	8	10	
BMI	23.38	23.33	.949
History of smoking			.658
Yes	2	4	
No	16	14	
ASA			1
I	4	4	
II	11	11	
III	3	3	
Comorbidities			
Hypertension	5	3	.691
Diabetes mellitus	1	2	.22
Cardiovascular disease	3	4	1
Cerebrovascular disease	1	0	1
Neoadjuvant chemo radiotherapy	18	18	
Histological type			.457
Squamous cell carcinoma	12	14	
Adenocarcinoma	6	4	
Others			
Tumor location			.700
Upper	0	0	
Middle	4	5	
Lower	14	13	
Operation time (min)	366.67 (SD: 82.67)	403.89 (SD: 71.01)	.340
Ligation of TD	1	1	1
Chylothorax	0	1	.310
Postoperative hospital stay (days)	10.77	12.44	.339
Death	0	2, related to COVID 19 infection	

Abbreviations: ASA, American Society of Anesthesiologists; BMI, body mass index; icg, indocyanine green; TD, thoracic duct.

**FIGURE 1:**
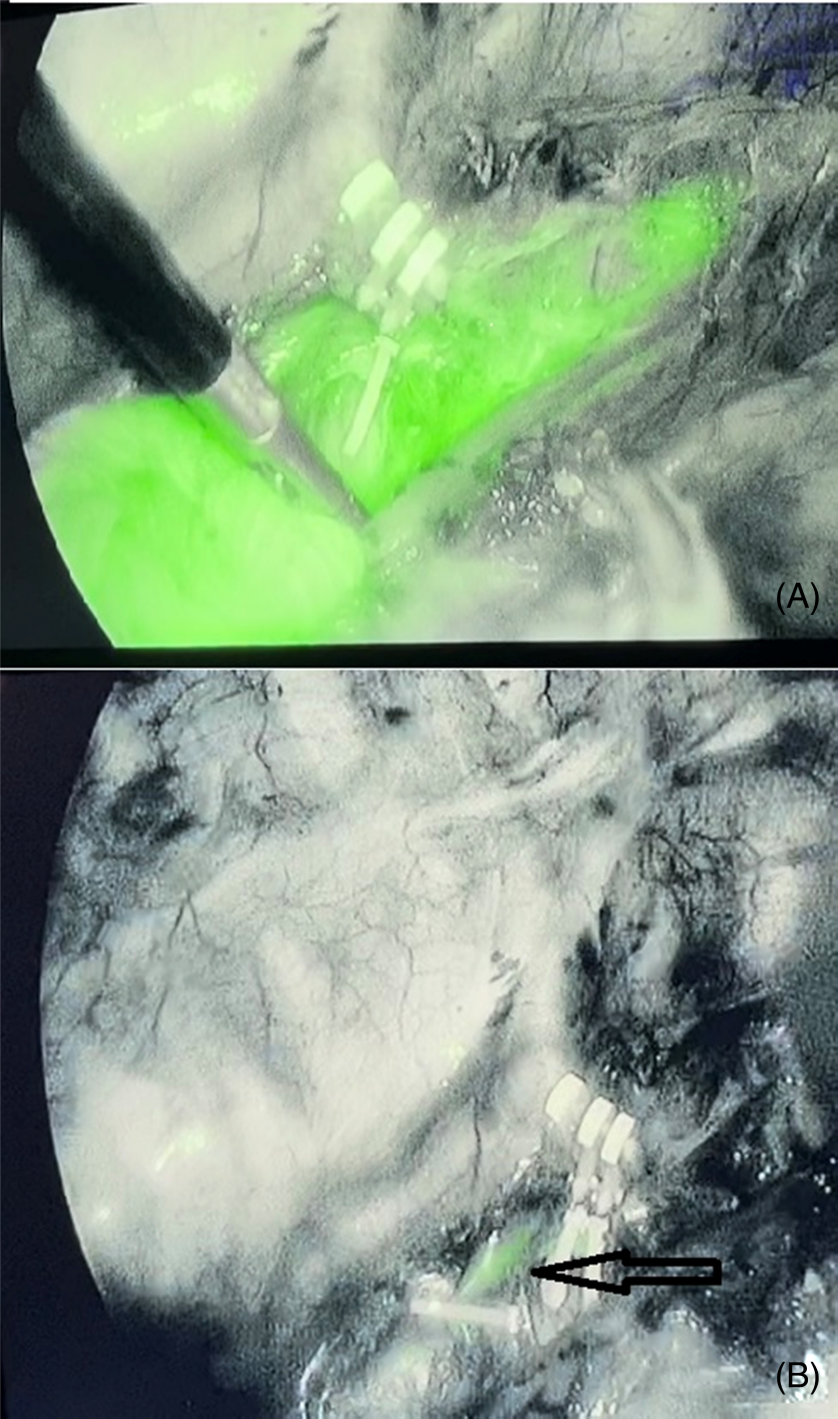
Injury of thoracic duct near azygous vein (A), ligation of injured part, arrow (B).

The median postoperative hospital stay was 10.77 (SD: 6.03) and 12.44 (SD: 4.10) days in ICG and non‐ICG groups, respectively. It was not significantly different. The mean operation time was 366.67 min (SD: 82.67) and 403.89 min (SD: 71.01) in ICG and non‐ICG groups, respectively. They were not significantly different. Each group had one (5.5%) TD ligation, in the ICG group near the azygous arc, and the non‐ICG group at the supradiaphragm. TD was ligated in the non‐ICG group for the oncologic purpose. In the ICG group injury was detected intraoperative, and ligation was performed at the site of injury (azygous arc; Figure 1). It was injured at the beginning of dissection due to its superficiality. NIR fluorescence with ICG confirmed efficient closure.

**FIGURE 2:**
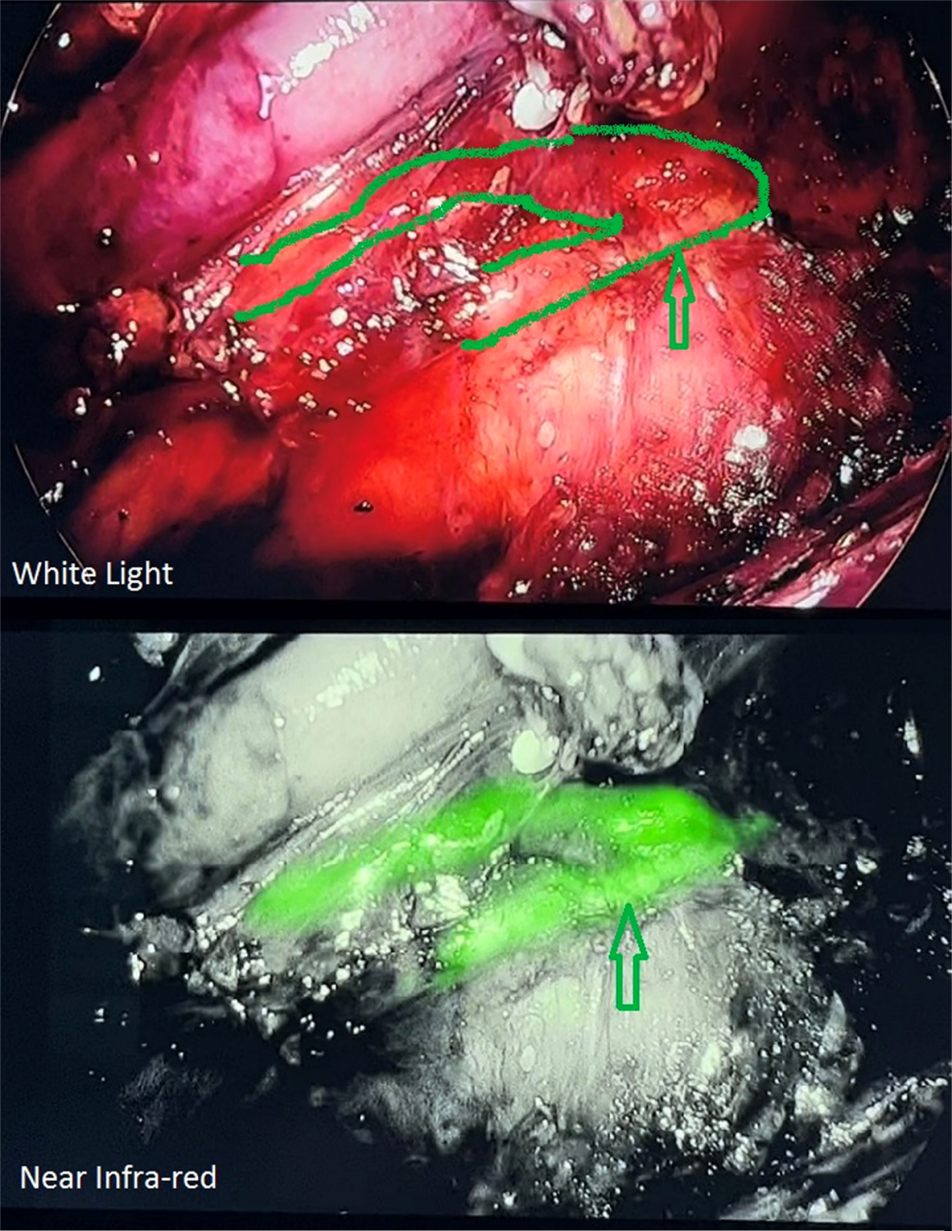
Intraoperative visualization of thoracic duct, arrow.

There were no adverse reactions or allergies to ICG or injection site pain. In all cases, the entire thoracic course of TD was visualized intraoperatively after a mean time of 81.39 min (SD: 29.69) from ICG injection to visualization (Figure 2 and Video S1) The Mean extra time for ICG injection was 11.94 min (SD: 3.88). This time included bilateral superficial inguinal lymph nodes injection under ultrasonography, turning off the lights in the operating room with the NIR imager, and recording video or taking appropriate photographs. Despite this additional time in the ICG group, the total duration of operation was less than the non‐ICG group, which may be due to the rapid progression of periesophageal dissection based on observing the TD. The Mean injection dose of ICG was 11.67 mg (SD: 2.4). In the ICG group, no patient suffered from CT. One patient in the non‐ICG group developed CT after surgery that was managed conservatively. According to Fisher's exact test, there was no significant association between CT development and ICG use, possibly due to the small sample size.

## 4 DISCUSSION

Esophagectomy is currently the only potentially curative treatment for esophageal cancer, but it is associated with high morbidity (50%) and mortality, even in high‐volume centers.^7^ In recent decades, minimally invasive procedures for esophagectomy have been developed to minimize surgical trauma and complication rates. Despite these technical improvements, the incidence of CT remains similar to that of open esophagectomy.^7^ CT is a rare but potentially life‐threatening complication that can lead to respiratory and metabolic problems.^8^, ^9^ Loss of chyle impairs wound healing due to protein loss and weight gain due to loss of fat. It also causes immunodeficiency due to the loss of lymphocytes and can lead to bacterial and fungal sepsis.^7^ It is important to diagnose and manage this condition early on to prevent further complications.

The treatment of chylorrhea remains a point of debate, and choosing between conservative therapy and surgery, as well as the timing of treatment, is still controversial.^10^ Conservative treatment involves the cessation of oral intake and can be successful depending on the underlying cause, with success rates ranging from 3% to 90%. Noninvasive or semi‐invasive procedures, such as TD closure or chest drain placement, also have varying success rates based on the etiology.^2^

Surgical therapy is another option, but it is only beneficial for patients fit for surgery and can have a morbidity and mortality rate of up to 25%. The success rate of surgical procedures ranges between 64% and 100%, but TD ligation itself is not without risk and should be avoided if possible.^2^

Clear visualization of the TD during surgical intervention can be difficult, especially in patients who have received neoadjuvant chemoradiotherapy. Reisenauer reported 97 postoperative CT after various surgical operations in 2018. In this study, 52 patients required ligation of TD, with a success rate of 85%. However, only 33 out of the 52 patients were able to have a clear visualization of the TD and selective ligation, while the other 19 had to undergo mass ligation.^11^

During esophagectomy, the probability of injury to the TD and its tributaries is high due to the variable anatomy of the TD.^3^, ^9^ Intraoperative identification of the TD can help overcome these challenges. However, despite several modalities that have been proposed to facilitate TD visualization, it cannot always be recognized, and in such cases, en‐mass ligation of the supradiaphragmatic region may be performed.^3^

Traditionally, lymphangiography has been considered the most reliable method for examining chylorrhea. However, it is a difficult procedure that involves the insertion of a tube into the lymphatic channels and can produce negative side effects like local tissue damage, fat embolism to the lungs, allergic reactions, or aggravation of lymphedema due to the contrast medium.^10^

There are other methods available to trace the leakage of chyle during surgery, such as feeding whole milk orally or nasally, CT lymphography, or methylene blue injection. However, these methods have some drawbacks such as low contrast, low reproducibility, interference with the surgical field, and mediastinal fat coverage.^12^

There are currently no standard and effective intraoperative diagnostic tools available for TD visualization. However, our team has utilized ICG fluorescence to precisely identify the TD in real‐time. ICG is an FDA‐approved fluorescent dye that has been used clinically since the 1960s.^2^, ^13^ It is generally considered to be a safe drug with minimal side effects, although it should be used with caution in patients with iodine allergies due to its sodium iodide content.^14^ Severe side effects of ICG administration may include anaphylactic reactions, hypotension, tachycardia, dyspnea, and urticaria, particularly in patients with chronic renal dysfunction.^10^ ICG was originally used as a fluorescence dye in angiography and can also be used in lymphography for identifying lymphatic tissue due to its strong fluorescence properties. One of the key benefits of using ICG is that it does not disrupt the surgical field or interfere with the resolution of the operation.^15^

ICG is a fluorescent dye that can bind to proteins in the blood and lymphatics, allowing these vessels to be visualized with NIR light.^10^ However, some tissue dissection is necessary before fluorescence can be seen due to low fluorescence penetration through tissues.^16^ When exposed to NIR light, ICG generates fluorescence of 800–850 nm wavelength. Intraoperative NIR imaging with ICG is a high‐quality imaging technique that may help surgeons perform safe dissection while receiving continuous anatomic feedback.^9^ As a result, it may eliminate the need for prophylactic TD ligation, which can lead to several postoperative complications.^2^ A systematic review conducted by Yiyan et al.^17^ concluded that there is no evidence to suggest a reduction of postoperative CT with the prophylactic TD ligation.

Ashitate et al.^15^ have demonstrated that ICG is the most effective lymphatic tracer among several NIR dyes for TD imaging. In animal models, they were able to observe normal TD and collateral flow, injuries, and repair animal models during both open and video‐assisted thoracoscopic surgery.

This novel application of NIR imaging may be effective in TD identification to assist fistula closure, as most chylous fistulas require open surgical ligation. Kamiya et al.^18^ reported the first experience with ICG usage for postoperative CT management in 2009. They presented a case of CT after an esophagectomy, where the patient had a right thoracotomy on the 28th postoperative day. During the exploration of the thorax, the exact site of the fistula was not identified, but it was detected only through ICG fluorescence images. Kaburagi et al.^19^ and Matsutani et al.^10^ also described two similar cases of CL after esophagectomy. Few other studies reported successful usage of this technique for postoperative CT management after lung surgery, pediatric surgery of esophageal atresia, Norwood surgery, and TEF operation.^3^, ^4^, ^12^

In their study, Yang et al.^9^ administered ICG subcutaneously (0.2 mg/kg) around 30 min before surgery in the bilateral inguinal region. They utilized real‐time fluorescence lymphography to identify the fistulas and the main trunk of the TD and then double‐ligated them with the help of the fluorescence guidance. According to various studies, ICG can be injected into the small bowel mesentery, inguinal subcutaneous region, and superficial inguinal lymph nodes.^7^, ^8^, ^20^ However, for this particular study, the researchers injected bilateral superficial inguinal lymph nodes while using ultrasonography.

Vershney et al.^8^ conducted a study that demonstrated the high effectiveness of administering ICG to the inguinal lymph nodes in describing the course of the TD during thoracoscopic esophagectomy and reducing the risk of postoperative CT. This method has also been successful in detecting the leakage point in patients with idiopathic recurrent CT.^5^

Table 2 summarizes the four studies that evaluated the intraoperative identification of TD using ICG during esophagectomy. This study is the first to compare the results with the same procedure without ICG usage. In this study, the TD and its branches were visible in all patients, as in the studies by Varshney et al. and Barbato et al. However, Vecchiato et al. identified TD in 95% of cases and Barnes et al. in 80% of cases. Identifying the TD in our study helped the surgeon navigate the dissection around the esophagus more quickly.

**TABLE 2:** Studies evaluating the intraoperative identification of thoracic duct using indocyanine green (ICG).

	Study	Number of patients	Procedure	ICG insertion site	Dose (mg)	TD visualized (%)	Time for visualization	Ligation of TD	TDI	Postoperative chylothorax
1	Vecchiato et al.^6^	20	TTE status NACRT	Inguinal LN	0.5 mg/kg	19 (95%)	35–80 min	2	0	0
2	Barnes et al.^7^	20	TTE (thoracoscopic and Open)	Enteral route or small bowel mesentery	3—6.2	16 (80%)	10–185 min	4	4	1
3	Barbato et al.^8^	18	Robotic Ivor‐Lewis esophagectomy	Subcutaneously in the inguinal region	0.5 mg/kg	18 (100%)	18–24 h	18 Routine prophylactic ligation done	1	0
4	Varshney et al.	21	Robotic and Thoracoscopic McKeown esophagectomy status NACRT/NACT	Inguinal LN	2.5–7.5	21 (100%)	30–80 min	3	1	0
5	This study	18	Minimal invasive TTE, NACRT	Inguinal LN	10 mg	17 (100%)		1 (ligation of injured part)	1	0

Abbreviations: ICG, indocyanine green; LN, lymph node. min: minute; mg, milligram; NACT, neoadjuvant chemotherapy; NACRT, neoadjuvant chemo radiotherapy; TD, thoracic duct; TDI, thoracic duct injury. TTE, transthoracic esophagectomy.

As a result, the total procedure time was shorter in the ICG group than in the non‐ICG group, although the differences were insignificant. One case had a TD injury during the dissection of the azygous vein at its arc before the visualization of TD. After switching to NIR mode, extravasation of fluorescence dye from the fluorescent tubal duct was seen, and a TD injury was diagnosed. The injury site was ligated with suture clips, and extravasation was stopped. Four patients in the study conducted by Barnes et al. had TDI, while no patient in the study by Vecchiato et al. suffered from it. In the studies by Barbato et al. and Varshney et al., as well as in our own study, one patient had TDI. On the other hand, no patients in our study experienced CT, which was also the case in the studies by Vecchiato et al., Barbato et al., and Varshney et al. However, one patient in Barnes et al. did suffer from postoperative CT.

## 5 CONCLUSION

Esophageal surgery is a complex process that poses significant risks to patients. Surgeons are now exploring new tools to help mitigate these challenges. It is important to note that while intraoperative fluorescence imaging shows promise for improving perioperative and oncological outcomes, further research is necessary to confirm its benefits. To establish this technique's effectiveness in preventing postoperative CT, larger sample sizes will need to be used in future studies. Additionally, it is worth noting that this study is limited by its small sample size and lack of randomization. To better demonstrate the usefulness of this approach, further randomized clinical trials should be performed.

## AUTHOR CONTRIBUTIONS

**Habibollah Mahmoodzadeh:** Conceptualization (lead); data curation (supporting); formal analysis (supporting); investigation (lead); methodology (lead); project administration (lead); resources (supporting); software (supporting); supervision (lead); validation (lead); visualization (lead); writing – original draft (supporting); writing – review and editing (supporting). **Athena Farahzadi:** Conceptualization (lead); data curation (lead); formal analysis (lead); investigation (lead); methodology (lead); project administration (lead); resources (lead); software (lead); supervision (lead); validation (lead); writing – original draft (lead); writing – review and editing (lead). **Ramesh Omranipour:** Conceptualization (supporting); supervision (supporting); visualization (supporting); writing – original draft (supporting); writing – review and editing (supporting). **Iraj Harirchi:** Writing – original draft (supporting); writing – review and editing (supporting). **Amir Mohsen Jalaeefar:** Writing – original draft (supporting); writing – review and editing (supporting). **Mohammad Shirkhoda:** Writing – original draft (supporting); writing – review and editing (supporting). **Seyed Rouhollah Miri:** Writing – original draft (supporting); writing – review and editing (supporting). **Farimah Hadjilooei:** Writing – original draft (supporting); writing – review and editing (supporting).

## CONFLICT OF INTEREST STATEMENT

Prof. Habibollah Mahmoodzadeh, Dr. Athena Farahzadi, Prof. Ramesh Omranipour, Prof. Iraj Harirchi, Dr. Amir Mohsen Jalayifar, Dr. Mohammad Shirkhoda, Dr. Ruohollah Miri, and Dr. Farimah Hadjilooei have no conflicts of interest to disclose.

## ETHICS STATEMENT

Our Ethical Committee of Tehran University of Medical Science approved the use of NIR fluorescence imaging with ICG. All patients signed written informed consent forms.

## INFORMED CONSENT STATEMENT

Written informed consent was obtained from the patient for publication of this report in accordance with the journal's guidelines for patient informed consent.

## Supporting information


**Video S1:** Visualization of the thoracic duct with indocyanine green fluorescence during minimally invasive esophagectomy.

## Data Availability

The authors of this study have made the data available upon request from the corresponding author. Due to privacy or ethical restrictions, the data are not publicly available.
